# Allele-specific enhancers mediate associations between *LCAT* and *ABCA1* polymorphisms and HDL metabolism

**DOI:** 10.1371/journal.pone.0215911

**Published:** 2019-04-30

**Authors:** Alicia D. Howard, Xiaochun Wang, Megana Prasad, Avinash Das Sahu, Radhouane Aniba, Michael Miller, Sridhar Hannenhalli, Yen-Pei Christy Chang

**Affiliations:** 1 Division of Endocrinology, Nutrition, and Diabetes, Department of Medicine, University of Maryland School of Medicine, Baltimore, Maryland, United States of America; 2 Center for Bioinformatics and Computational Biology, University of Maryland, College Park, Maryland, United States of America; 3 Center for Preventive Cardiology, Department of Medicine, University of Maryland School of Medicine, Baltimore, MD, United States of America; University of Iceland, ICELAND

## Abstract

For most complex traits, the majority of SNPs identified through genome-wide association studies (GWAS) reside within noncoding regions that have no known function. However, these regions are enriched for the regulatory enhancers specific to the cells relevant to the specific trait. Indeed, many of the GWAS loci that have been functionally characterized lie within enhancers that regulate expression levels of key genes. In order to identify polymorphisms with potential allele-specific regulatory effects, we developed a bioinformatics pipeline that harnesses epigenetic signatures as well as transcription factor (TF) binding motifs to identify putative enhancers containing a SNP with potential allele-specific TF binding in linkage disequilibrium (LD) with a GWAS-identified SNP. We applied the approach to GWAS findings for blood lipids, revealing 7 putative enhancers harboring associated SNPs, 3 of which lie within the introns of *LCAT* and *ABCA1*, genes that play crucial roles in cholesterol biogenesis and lipoprotein metabolism. All 3 enhancers demonstrated allele-specific *in vitro* regulatory activity in liver-derived cell lines. We demonstrated that these putative enhancers are in close physical proximity to the promoters of their respective genes, *in situ*, likely through chromatin looping. In addition, the associated alleles altered the likelihood of transcription activator STAT3 binding. Our results demonstrate that through our approach, the LD blocks that contain GWAS signals, often hundreds of kilobases in size with multiple SNPs serving as statistical proxies to the true functional site, can provide an experimentally testable hypothesis for the underlying regulatory mechanism linking genetic variants to complex traits.

## Introduction

Genome-wide association studies (GWAS) have thus far identified polymorphic loci underlying hundreds of diseases and traits. For blood lipid levels alone, approximately 180 loci have been reported for traits such as low density lipoprotein cholesterol (LDL-C), high density lipoprotein cholesterol (HDL-C), and total cholesterol (TC) and triglycerides (TG) [1–9]. With ever-increasing sample sizes from multiple studies of trans-ethnic participants, meta-analyses of previous GWAS, and even GWAS based on electronic health records [10], an increasing proportion of the variability and estimated heritability of these traits is accounted for by these loci, collectively. However, the molecular mechanism underlying these associations is largely unknown.

Most of the GWAS signals reside in non-coding regions and consist of multiple SNPs in strong or complete linkage disequilibrium (LD). Establishing the causal variant(s) and determining the molecular mechanisms linking the variants to the trait are among the fundamental challenges of such studies. GWAS-identified loci are enriched for the enhancers of relevant cell types [11, 12]. In fact, the few blood lipid associated loci near known causal genes lie within regulatory enhancers exhibiting allele-specific transcriptional activities [13–16]. As the human genome has become richly annotated with tissue- and cell-type specific epigenetic signatures shared by regulatory elements [11, 17], identifying the causal regulatory variant underlying a genetic signal is becoming increasingly feasible.

Here we identified 7 SNPs within putative enhancers and are in LD with blood lipids associated SNPs. Reporter assay revealed 3 of the enhancers to have allele-specific regulatory effect. Interestingly, the three validated SNPs reside in the introns of *ABCA1* and *LCAT*, which encode a critical transporter and a pivotal enzyme in the HDL metabolic pathway, respectively. Furthermore, the SNPs with *in vitro* regulatory activity showed physical proximity to the target gene promoter and the expected transcription factor-enhancer interaction *in situ*.

## Materials and methods

### Enhancer SNPs prediction

First, we started with GWAS signals provided by the NHGRI GWAS Catalog, which is a collection of manually curated, literature-derived genome-wide association studies, including only studies that assayed at least 100,000 SNPs and SNP-trait associations with p-values < 1.0 x 10^−5^ [18]. To enrich for signals with larger effect sizes, we focused on earlier GWAS studies (2005–2011), which consisted of 5,903 GWAS signals with 4,789 unique SNPs, related to 342 diseases or traits from 745 GWAS publications. Second, we filtered the diseases/traits to focus on those related to blood lipids and GWAS signals from European or European-American studies resulting in 260 GWAS SNPs or 140 independent signals. An independent signal is defined as a cluster of associated SNPs in strong LD located >100 kb away from other GWAS signals. The 9 blood lipid-related traits are LDL, HDL, TG, HDL-TG, total cholesterol, cholesterol, lipid metabolism, TG-BP, response to statin. Most of the 260 SNPs associated with blood lipids are in intronic and intergenic regions (43.5% and 35.4%, respectively). Locations of these 260 GWAS SNPs based on information from ANNOVAR [19], is provided in S1 Table. Third, we extended the SNPs reported to include strongly correlated SNPs based on HapMap CEU data (r^2^ ≥ 0.3), resulting in 6,230 SNPs. Finally, for each SNP in this extended set, we examined a 500 bp region, centered on the SNP and we applied our previously published machine learning model for enhancer prediction based on experimentally derived epigenetic signatures of known enhancers such as chromatin status, histone modification and transcription factor binding derived from hepatoma cells HepG2 [20]. We applied the same cutoff that has a cross-validation accuracy of ~90% based on 415 experimentally validated heart-specific enhancers in our published work [20], resulting in a set of 34 unique SNPs located in putative enhancers that we refer to as enhancer SNPs or eSNPs (available upon request). We further selected eSNPs that are in strong LD with the GWAS SNPs (r^2^≥0.8 and D’≥ 0.9). Because we are primarily interested in enhancers, we prioritized eSNPs that are located in the 5’, 3’, intronic, and intergenic regions and not those located near transcription start sites and promoter. For experimental validation, we focused on 7 eSNPs (Table 1). The SNP filtering process is detailed in S1 Fig.

**Table 1:** Putative eSNPs in LD with trait-associated GWAS SNPs.

Chr	eSNP	GWAS SNP	r^2^	Trait(s)	p-value	Reported Causal Gene(s)	Nearest Gene(s)	Reference(s)
1	rs17315646	rs10489615	0.97	HDL	4x10^-9^	*GALNT2*	*GALNT2*	[6]
		rs2144300	1	HDL	3x10^-14^			[2]
				TG	8x10^-7^			[2]
		rs4846914	1	HDL	2x10^-13^			[1],[4]
				TG	7x10^-15^			[1]
9	rs2575875	rs4149268	0.96	HDL	1x10^-10^	*ABCA1*	*ABCA1*	[2]
9	rs3847301	rs3890182	0.81	HDL	3x10^-10^	*ABCA1*	*ABCA1*	[1],[6]
		rs3905000	0.81	HDL	9x10^-13^			[3]
9	rs643531	rs643531	1	HDL	7x10^-9^	*TTC39B*	*TTC39B*	[6]
11	rs12287066	rs12272004	0.83	TC	7x10^-7^	*APOA1-C3-A4-A5*	*APOA5*	[3]
				LDL	5x10^-13^			[3]
				TG	5x10^-13^			[3]
		rs12286037	1	TG	1x10^-26^			[2]
		rs28927680	1	TG	2x10^-17^			[1]
16	rs1109166	rs12449157	0.94	HDL	2x10^-7^	*GFOD2*, *LCAT*	*LCAT*, *SLC12A4*	[6]
19	rs2075650	rs2075650	1	TC	3x10^-19^	*TOMM4*, *APOE*	*TOMM4*, *APOE*	[3]

r^2^ is based on HapMap CEU.p-values from the original GWAS studies.

### Plasmid construction

Sequences encompassing putative enhancers were PCR-amplified from human genomic DNA, using the primers listed in S3 Table, and cloned into the pCR8/GW/TOPO Gateway entry vector (Invitrogen). The LR Clonase system (Invitrogen) was used to generate expression clones by recombination between the entry vector and a pGL3 vector modified to incorporate Gateway sequence within the SmaI site. Plasmids containing a putative with the alternate SNP allele were generated by mutagenesis according to the QuikChange Site-Directed Mutagenesis Kit (Agilent Technologies). The boundaries of enhancers were determined by available DNase I hypersensitive sites and histone modifications.

Some of the GWAS SNPs and putative eSNPs (rs10489615, rs3890182, rs28927680 and rs17315646) in our study are reportedly tri-allelic. However, in all cases the least common alleles are found rarely, due to either extremely low allele frequencies or genotyping error. For example, rs10489615 is a A/G SNP in 86 out of 87 dbSNP submissions and a A/C SNP in one submission. Since these rare alleles, even if real, are unlikely drive the association signals, our experimental validation will focus only on the 2 common alleles that are considered RefSNP alleles by dbSNP.

### Cell culture

Human HepG2, Huh-7, and HEK293 cells used for reporter assays were grown at 37°C with 5% CO_2_ in Dulbecco's Modified Eagle Medium supplemented with 10% fetal bovine serum (FBS). For immunoblot and ChIP HepG2 and Huh-7 cells were grown in 10% FBS-supplemented Eagle’s minimum essential medium (MEM) and Dulbecco's Modified Eagle Medium, respectively.

### Reporter assays

Approximately 1x10^5^ cells were seeded in 24-well plates 24 hours prior to transfection. Constructs containing putative enhancers (200ng) were co-transfected with 2ng of the phRL-SV40 Renilla control vector, in triplicate, in the presence of Opti-MEM and Lipofectamine 2000 (Invitrogen) per manufacturer's recommendations. Adherent cells were incubated with the DNA–Lipofectamine complexes for 24 hours, rinsed with 1X PBS and lysed with 1X Passive Lysis Buffer (Promega).

Dual luciferase assays were performed in triplicate following standard protocols for the Dual-Glo Luciferase Assay System (Promega, E1910) using 50X- or 25X dilution of the Stop and Glo substrate reagent. Luciferase counts were read on the Perkin-Elmer Victor3 Plate reader. The normalized luciferase activity was determined by dividing the raw firefly luciferase value by the raw Renilla luciferase value. Relative luciferase activity was then calculated by dividing the normalized luciferase values for the enhancer construct by that of the normalized luciferase of the enhancer-less pGL3 promoter vector (Promega, E1761). A previously reported liver enhancer served as the positive control [21]. Putative enhancers, in which at least one allele demonstrated higher activity than the positive control, were carried forward for additional validation. Enhancer activities of the two alleles were compared using student’s t test.

### Electrophoretic mobility shift assay (EMSA)

Nuclear extracts were prepared from Huh-7 cells using the NE-PER Nuclear and Cytoplasmic Extraction Kit (Thermo Fisher, 78833) according to the manufacturer's instructions. Oligonucleotide probe sequences consisted of 33-bp centered on the rs1109166, rs2575875, and rs3847301 SNP alleles and are listed in S4 Table. Sense and antisense strands were labeled using the Biotin 3' End DNA Labeling Kit (Thermo Fisher, 89818). Huh-7 nuclear extract (3–5 ug) was mixed with 200 fmol of biotinylated probe, 50 ng/μL Poly (dI•dC) non-specific competitor DNA and 5X binding buffer (Thermo Fisher, 20148A) supplemented with 5% glycerol and 1mM MgCl_2_. Binding reactions were incubated at room temperature for 20 minutes, and electrophoresed on a 6% non-denaturing polyacrylamide gel (29:1) in cold 0.5X TBE buffer. For competition reactions nuclear extracts were incubated with 100-fold excess of unlabeled, oligonucleotide probe prior to addition of the labeled probe. STAT1 [22], STAT3 [23], and HNF4A [24] competitor oligos were obtained from published literature.

### Chromosome conformation capture

Chromosome conformation capture (3C) was performed similarly to the protocol described by [25] Huh-7 cells were harvested by treatment with 0.25% (w/v) trypsin-EDTA at 37°C for 5 minutes. Approximately 1x10^7^ cells were resuspended in PBS with 10% FBS, then cross-linked at room temperature for 10 minutes by addition of formaldehyde at 1% of the final volume. Crosslinking was quenched by addition of glycine to the final concentration of 0.125 M. Cells were lysed in cold lysis buffer (10mM Tris-HCl, pH 8.0, 10mM NaCl, 0.2% NP-40, protease inhibitors) on ice for 15 minutes. Cells were pelleted at 4°C, then resuspended in 312 uL of 1.2X restriction enzyme buffer (NEB). 10 uL was removed for the undigested control. 38 uL of 1% SDS was added to the remaining nuclei. The mixture was incubated at 65°C for 10 minutes before addition of 44 uL of 10% Triton X-100. A 5μl aliquot was removed for the undigested control. Four hundred units of either MspI, NcoI, or PstI restriction enzyme was added to the remaining chromatin prior to overnight incubation at 37°C, with shaking. Restriction digestion was stopped by addition of 86 uL of 10% SDS and incubation at 65°C for 30 minutes, with shaking. A 5μl aliquot was then removed for the digested but no ligated control. Cohesive ends were ligated using 3350 units of T4 DNA ligase (NEB, M0202S) for 4 hours at 16°C, then 30 minutes at room temperature under dilute conditions in 1X ligation buffer (10% Triton X-100, 1X Ligation buffer (NEB), and 10 mg/ml BSA). Three hundred units of proteinase K (NEB, P8107S) was added to the ligation mixture prior to reversal of crosslinks overnight at 65°C. The digested and undigested controls were incubated overnight at 65°C after addition of 500μl 1X restriction enzyme buffer and 20 units of proteinase K. Finally, DNAs were purified using phenol-chloroform extraction, and ethanol precipitation. The digested and undigested controls were resuspended in 60μl of 10mM Tris pH 8.0, while the 3C template was resuspended in 150μl of 10mM Tris pH8.0.

3C PCR primers were designed along the same strand and in the same orientation to accomplish specific amplification across 3C ligation junctions. Primers used to detect ligated fragments from potential SNP-promoter interactions are listed in S5 Table. Amplicons of sizes consistent with enhancer-promoter interaction were tested by restriction enzyme digestion and subsequently confirmed by sequencing.

### Transcription factor binding prediction

The vertebrate transcription factor binding site motifs from JASPAR CORE database [26] were used to predict allele-specific transcription factor-binding sites within a 15-mer sequence centered at the SNP. A SNP was deemed to have allele-specific binding if one of the alleles achieved a score above 60 percentile and the other allele below 60th percentile.

### Western blot

Nuclear and cytoplasmic fractions were isolated from untreated, acetic acid-treated, recombinant human IL-6 (rhIL-6)-stimulated Huh-7 and HepG2 cells using the NE-PER kit. Protein concentration was quantified by BCA assay (Pierce) and approximately 5ug of nuclear protein were denatured and separated by SDS-PAGE and transferred to PVDF membrane. Membranes were blocked in 5% BSA-TBST buffer followed by incubation with an antibody directed against STAT3, phosphorylated at tyrosine 705, (anti-pSTAT3-Y705, Cell Signaling Technology, D3A7). Membranes were stripped and re-probed for total STAT3 (Santa-Cruz, sc-482X) and HDAC1 (BioLegend, 607401) using 1:2000 and 1:1000 dilutions, respectively. Relative levels of induced phospho-STAT3 protein were determined by band densitometry (Alpha Innotech).

### Chromatin immunoprecipitation

The chromatin immunoprecipitation (ChIP) assay was carried out essentially as described in the Pierce Magnetic ChIP kit protocol (Thermo Fisher, 26157). Approximately 4x10^6^ Huh-7 and HepG2 cells were harvested after 30-minute treatment with recombinant human IL-6 (rhIL-6, 100 ng) or acetic acid (100 mM). Protein-DNA complexes were incubated in the presence of 5ug of anti-STAT3 antibody (Santa Cruz, sc-482X) or rabbit IgG (Santa Cruz, sc-2027) overnight. Immune complexes were captured using magnetic beads for 2 hours, followed by elution and purification of DNAs. Enrichment was determined by quantitative PCR using LightCycler 480 SYBR Green I Master mix (Roche, 04707516001) and the control GAPDH primers (included in the kit) listed in S6 Table. Relative enrichment was calculated by normalizing the Cp values of the purified immunoprecipitated DNAs to respective input controls (10% of total chromatin), followed by calculating the fold-enrichment of the antibody-immunoprecipitated DNA over the non-specific rabbit IgG control. Quantitative PCR results were compared using student’s t test.

## Results

### Linking SNPs associated with cholesterol metabolism to putative liver enhancers

Starting with the curated GWAS SNPs, our *in silico* pipeline extended this set of SNPs to include other SNPs in LD. Then we identified those that are likely to lie within an enhancer based on our previously developed machine learning model for enhancer prediction based on experimentally-derived epigenetic signatures of known enhancers [20]. We performed such analyses using GWAS hits related to blood lipids and epigenetic data from a liver-derived cell line, HepG2 (available through ENCODE). From 140 independent GWAS signals, we identified 34 putative enhancers with eSNPs (34/140 = 24%). Of these 34 eSNPs, 16 are in strong LD with the GWAS SNPs (r^2^> 0.8) and are likely the causal variants driving the association with one or more of the 9 traits related to cholesterol metabolism. Because of the similarity between the epigenetic signature of promoters and enhancers, and our primary interest in enhancer SNPs, we focused on 7 of these 16 putative eSNPs located outside of the promoter region for detailed experimental validation and characterization (see Methods and S1 and S2 Figs for details). These 7 putative eSNPs are in strong LD with replicated blood-lipid GWAS signals (Table 1) from multiple GWAS and subsequent meta-analyses. The effect sizes and significance levels from the Global Lipids Genetics Consortium, are shown in S2 Table.

### Putative enhancers within and near 2 HDL-C genes demonstrated *in vitro* enhancer activity

**Fig 1:**
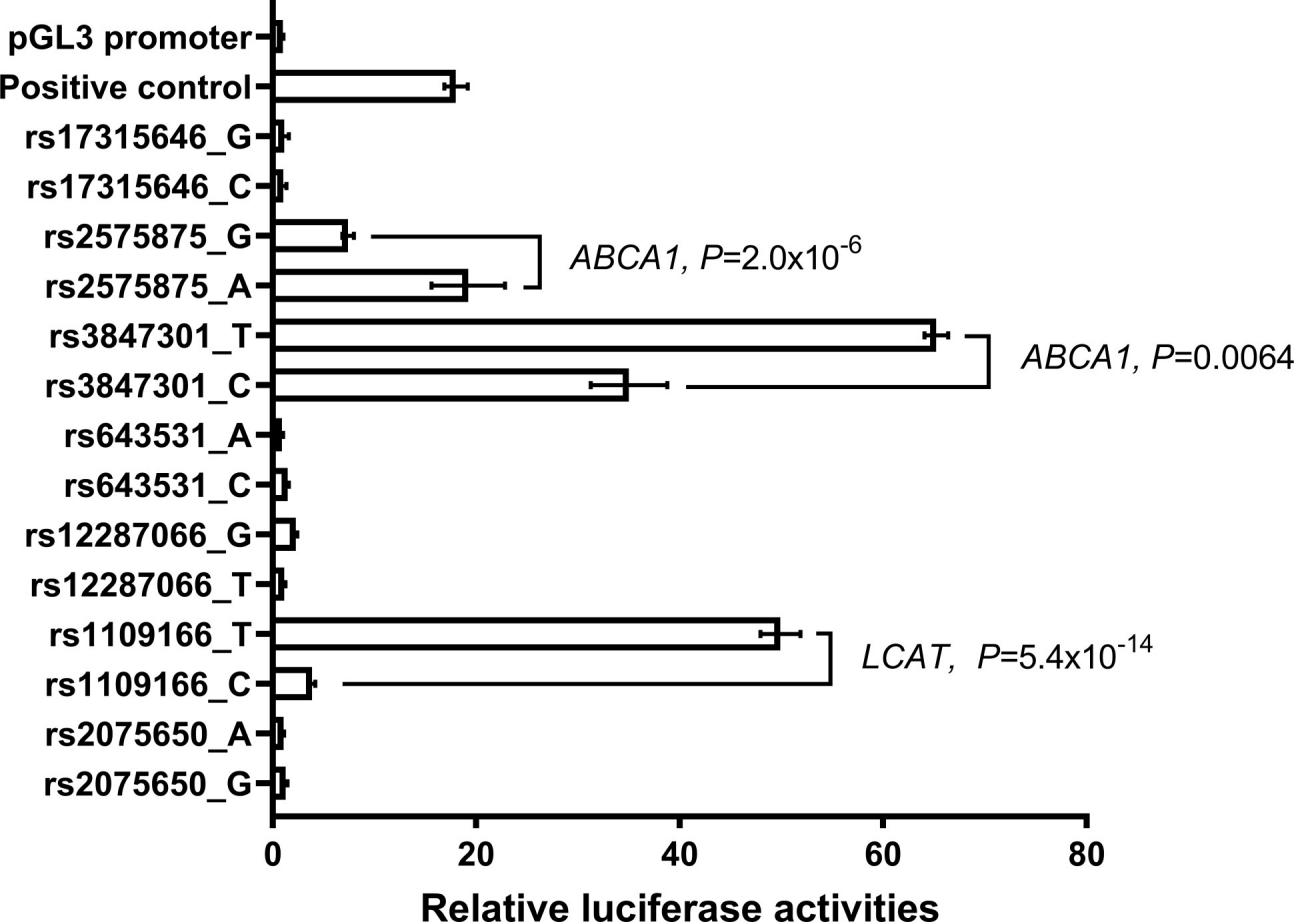
*In vitro* validation of enhancer activity. Putative enhancers with rs1109166, rs2575875, and rs3847301 showed *in vitro* enhancer activity, with at least 1 allele, higher or comparable to that of a known liver enhancer [21]. A construct with a minimal promoter but no enhancer (pGL3 promoter) is used to establish baseline transcription activity. Gene names (ABCA1 and LCAT) near the HDL-C associated eSNPs are shown.

To determine *in vitro* allele-specific enhancer activity, we cloned the regions containing the 7 eSNPs into a luciferase expression reporter vector under the control of a minimal promoter. Enhancer activity in a hepatoma-derived cell line, Huh-7, was measured as relative luciferase activities of 14 constructs (Fig 1). When compared to the activity of a known enhancer [21], three putative enhancers containing eSNPs rs2575875, rs3847301 and rs1109166, had at least one allele that demonstrated enhancer activity (19, 65, and 50 times higher than the negative control, respectively). Similar, but lower enhancer activities (2–5 times higher than the negative control), were also detected in another hepatoma-derived cell line, HepG2, known to have lower transfection efficiency (S3 Fig). Interestingly, some of the putative enhancers that did not show enhancer activity in liver-related cells did so in another cell line, HEK293 (S3 Fig).

The 3 putative enhancers containing eSNPs rs2575875, rs3847301 and rs1109166 are located within or near 2 known HDL regulating genes, *ABCA1* and *LCAT* (Table 1). SNPs rs2575875 and rs3847301 track independent association signals (r^2^<0.3 in all HapMap samples) and are located in intron 2 and intron 3 of *ABCA1*, respectively. SNP rs1109166 is located in intron 1 of *LCAT*. While these 2 genes play well-established roles in HDL metabolism, and multiple GWAS studies have identified SNPs associated with HDL-C levels, the molecular mechanisms underlying these associations have not been previously reported.

### Potential promoter-enhancer interaction detected by 3C

**Fig 2:**
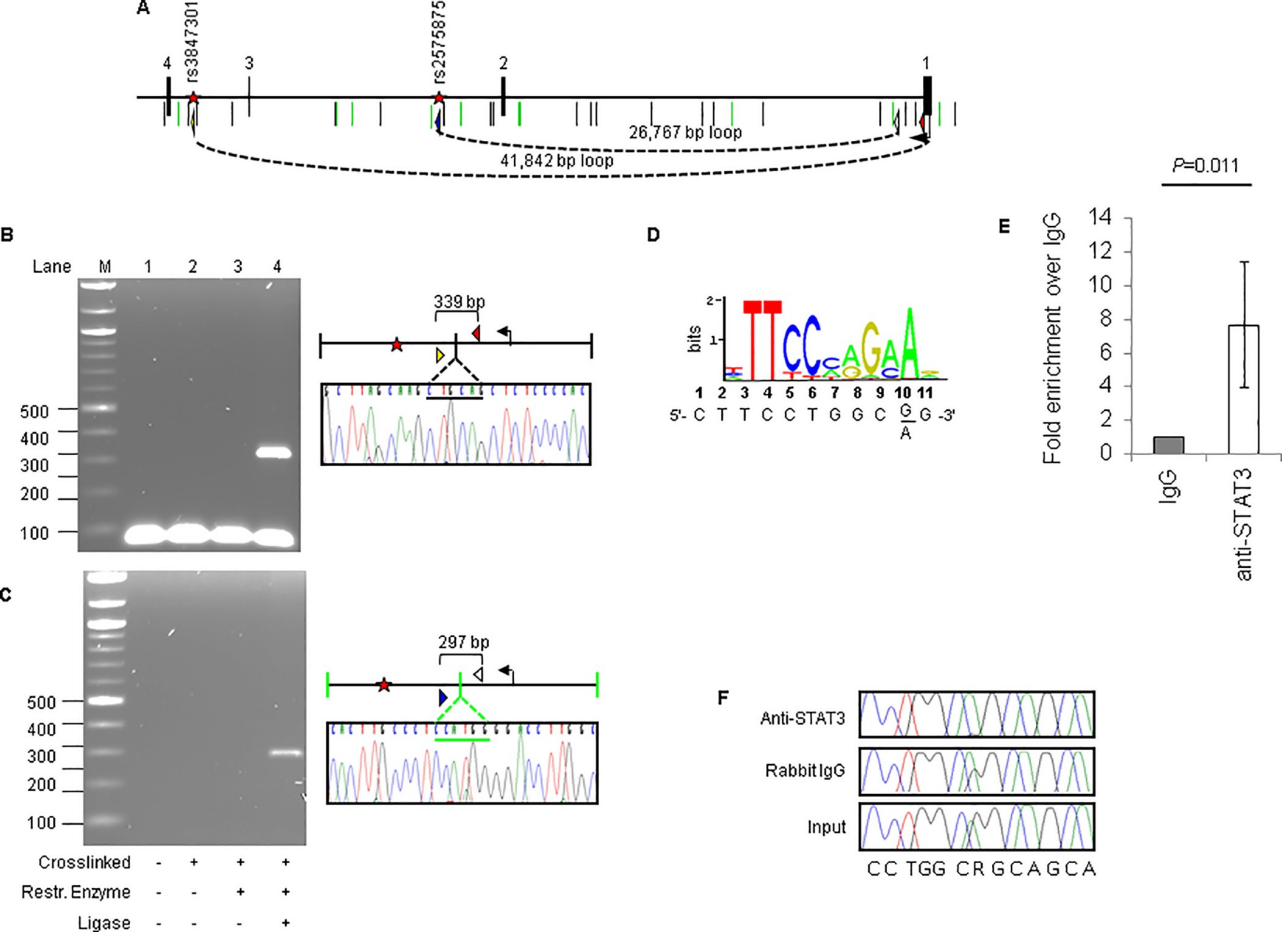
Chromatin interactions between eSNPs and the *ABCA1* promoter. (A) The region containing the first 4 exons of ABCA1. Black and green vertical lines depict PstI and NcoI sites respectively. The location of the eSNPs are shown as red stars. Primers used to detect 3C interactions are showed as colored triangles with the apex indicating direction of amplification. Possible chromatin loops between eSNPs and ABCA1 promoter are indicated by dash lines and the distances between the 2 restriction fragments. (B) 3C interactions between ABCA1 promoter and eSNP rs3847301. Negative controls (experiments performed without cross-linked DNA, restriction enzyme or ligase) are also shown. 3C product is confirmed by Sanger sequencing. PstI site is indicated by black underline. Transcription start site is indicated by a horizontal arrow. (C) 3C interactions between ABCA1 promoter and eSNP rs2575875. NcoI site is indicated by green underline. (D) The A allele of eSNP rs2575875 create a predicted STAT3 binding site, as shown by position weight matrix of STAT3 (JASPAR). Sequence flanking the SNP is shown below the weight matrix. (E) Quantitative PCR following chromatin immunoprecipitation with anti-STAT3 antibody in HepG2 cells showing an 8-fold enrichment of the sequence containing rs2575875. (F) Allele-specific enrichment of rs2575875 A allele is demonstrated by sequencing HepG2 DNA before treating cells with anti-STAT3 antibody (input, showing that HepG2 is normally heterozygous A/G at this site), with a control antibody (rabbit IgG) and with anti-STAT3. DNA recovered following ChIP with anti-STAT3 contains almost exclusively the A is allele.

**Fig 3:**
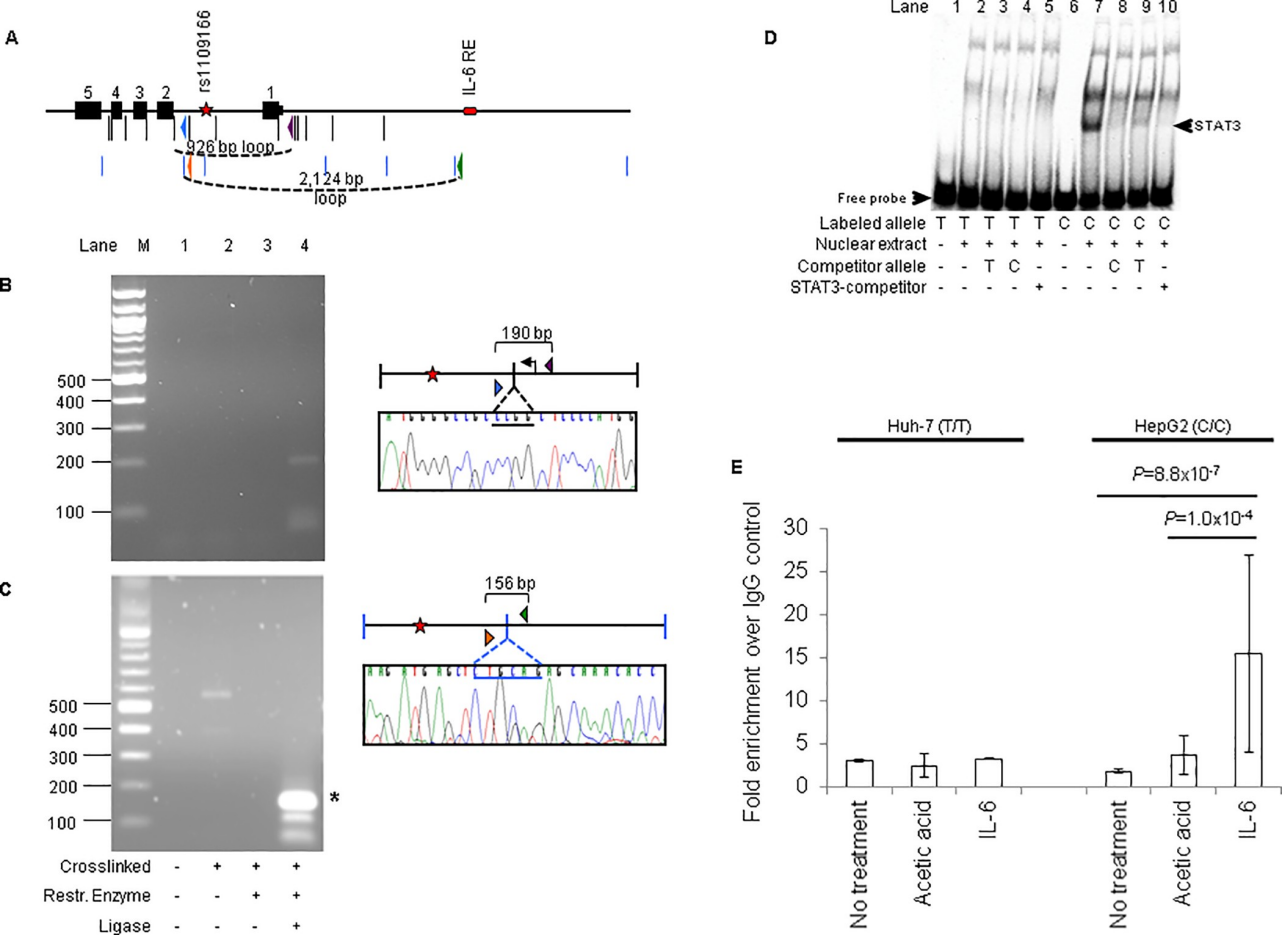
Chromatin interactions between rs1109166 and the *LCAT* promoter and an IL-6 response element. (A) The region containing the 5’ region and the first 5 exons of the *LCAT*. Black and blue vertical lines depict MspI and PstI sites respectively. A previously characterized IL6-responsive element [27] is indicated by a red bar. Other symbols are described in Fig 2. (B) 3C interaction between the SNP-promoter fragments. (C) 3C interaction between the SNP SNP and IL-6- responsive element. Distal interactions were only detected in cross-linked chromatin, in the presence of ligase, and were confirmed by Sanger sequencing. (D) STAT3 competitor oligos compete away a rs1109166-C-specific band in EMSA (lane 10). (E) Chromatin immunoprecipitation shows enhanced STAT3-binding to rs1109166-C allele after cells are treated with IL-6.

Enhancers are thought to upregulate transcription by interacting with promoters through chromatin looping. to test if the putative enhancers containing eSNPs rs1109166, rs2575875, and rs3847301 and the relevant promoters participate in chromatin looping, we designed primers to detect such an interaction through chromatin conformation capture (3C); wherein non-adjacent fragments, bearing an enhancer and a promoter as many as several tens of kilobases apart, are ligated together to form a junction detectable by PCR. Chromatin from Huh-7 cells was cross-linked, digested and ligated. We found that rs2575875 and rs3847301, which are independently associated with HDL-C levels, demonstrated 3C interactions with the *ABCA1* promoter region (Fig 2A–2C). SNP rs1109166 interacts with not only the *LCAT* promoter, but also a published IL-6 responsive element that regulates *LCAT* through STAT3 binding (Fig 3A–3C) [27]. Although the enhancer containing rs1109166 overlaps another gene, *SLC12A4*, which encodes a potassium and chloride transporter with unknown function (S2 Fig), no 3C product was detected between rs1109166 and the *SLC12A4* promoter.

### ABCA1 enhancer containing SNP rs2575875 shows allele-specific binding to transcription activator STAT3

The A allele of eSNP rs2575875 was predicted to bind to STAT1/STAT3 based on motif obtained from JASPAR [26] (Fig 2D and S4 Fig). Oligonucleotides containing the A allele of this SNP, but not the G allele, bind nuclear protein(s) that can be competed away with oligonucleotides containing known STAT1 and STAT3 binding sequences (S4 Fig). In contrast, both alleles of eSNP rs3847301 are predicted to bind HNF4A and no such allele-specific binding of nuclear protein and oligonucleotides was observed (S5 Fig).

Interestingly, the interaction of ABCA1 and apoA-1 increases the phosphorylation of STAT3, but not STAT1, through JAK1 [28, 29]. ABCA1 contains two STAT3 docking sites located in its large cytosolic loops. Given such protein-protein interaction, we explored the possibility that STAT3 also activate *ABCA1* transcriptionally through STAT3-enhancer binding. Since HepG2 is heterozygous A/G at the rs2575875 site (Fig 2F, input panel), we experimentally validated this allele-specific interaction by performing chromatin immunoprecipitation (ChIP) with an antibody against STAT3. Incubation of HepG2 cellular lysate with the antibody enriched the chromatin region containing rs2575875 by approximately 8-fold (Fig 2E). Importantly, the chromatin recovered following immunoprecipitation contained almost exclusively the A allele (Fig 2F).

### LCAT enhancer containing SNP rs1109166 C allele binds STAT3 and binding is increased by IL6

A previous study has identified STAT3 binding IL-6 responsive element 1.5 kb upstream of the promoter of *LCAT* [27]. We show that our rs1109166 in the LCAT intronic enhancer interacts with the IL-6 responsive element (Fig 3A and 3C) [27]. We therefore tested whether the intronic enhancer participated in the IL-6 signaling pathway. First, we showed that the sequence flanking rs1109166 containing the C allele binds nuclear protein(s) that can be competed away with known STAT3-binding oligonucleotides (Fig 3D). Next, we took advantage of the fact that Huh-7 and HepG2 cells are homozygous T/T and C/C for rs1109166 (S6 Fig) respectively. We treated Huh-7 and HepG2 cells with IL-6 and showed an increase in the phosphorylation of STAT3 and, therefore, STAT3 activity in both cell lines (S7 Fig). Chromatin immunoprecipitation with anti-STAT3, however, showed enrichment of the SNP containing sequence only when HepG2 cells are treated with IL-6 (Fig 3E), suggesting allele-specific activity at rs1109166 upon IL-6 induction.

## Discussion

The most important finding in the present study is the identification of allele specific enhancers in *ABCA1* and *LCAT* that may effectively modulate HDL metabolism. Our study, like many others (recently reviewed and discussed by Catarino and Stark [30]), showed that not all predicted or known enhancers will consistently demonstrate enhanced transcription when tested ectopically in a reporter gene system. In this study, the putative enhancer near *GALNT2* containing rs17315646 did not demonstrate *in vitro* enhancer activity in Huh-7 and HepG2, even though the construct containing the G allele showed substantial (nearly 10-fold higher than the promoter-only construct) activity in HEK293 cells (S3 Fig). Another study [14] using a similar approach, found that a segment within the sequence we characterized that contains rs4846913, rs2144300, and rs6143660, and a nearby segment, containing SNP rs2281721, showed allele-specific increases in enhancer activity (S8 Fig). Sequences containing 2 SNPs, rs4846913 and rs2281721 also showed possible binding to nuclear proteins CEBPB and USF1, respectively [14]. In fact, this work demonstrated that multiple SNPs in this HDL-C associated locus act synergistically to influence *GALNT2* expression. In addition, these data point to the difficulties in predicting the boundaries of a regulatory element.

While our data show that the *LCAT* enhancer containing SNP rs1109166 interacts with STAT3 and the interaction is specific to the C allele (Fig 3D and 3E), the actual sequence flanking this SNP contains two weakly predicted STAT3 sites when containing the C allele and 1 weak STAT3 site when containing the T allele (S6 Fig). Therefore, it is unclear as to whether the STAT3-rs1109166 enhancer interaction is direct, is mediated through other transcription factor(s) with preference for the C allele or is through chromatin looping that involves the rs1109166 enhancer, the *LCAT* promoter, the IL-6 responsive element, and STAT3.

*LCAT* encodes lecithin-cholesterol acyltransferase, which catalyzes the esterification of free cholesterol to cholesteryl ester (CE) with delivery of CE to steroidogenic tissues in a process referred to as reverse cholesterol transport (RCT) [31]. Not only does LCAT play an important role in HDL metabolism, 2 defective *LCAT* alleles result in complete LCAT deficiency, a rare recessive disorder characterized by very low levels of HDL-C, progressive renal insufficiency and vascular disease [32]. Recently, the first combined kidney-liver transplant was performed in an effort to treat this condition [33].

ABCA1, also called the cholesterol efflux regulatory protein (CERP), encodes the ATP binding cassette transporter A1, involved in the export of cellular cholesterol from macrophages, representing the initial stage of RCT. Mutations in *ABCA1* may cause HDL-C deficiency and premature coronary artery disease (CAD) [34]. There is also suggestive evidence that common *ABCA1* promoter and coding variants (-565C>T, -470C>C, V825I) might be associated with age of symptom onset in CAD patients [35, 36].

Based on rare forms of LCAT and ABCA1 deficiency and associated low HDL-C levels, one might assume that the high HDL-C associated allele will track with higher *in vitro* transcriptional activity. However, this is not necessarily the case as evidenced by our data (Fig 1). For example, allele C of rs1109166 is in strong LD with allele G of GWAS hit rs12449157, yet there was significantly lower enhancer activity than allele T. This may not be surprising because HDL particles are heterogeneous in size, density and apolipoprotein composition and laboratory measurement of HDL-C does not capture the differences in *LCAT* expression. In fact, LCAT activity may not be correlated with HDL-C levels [37, 38].

Epidemiologically, elevated levels of HDL-C are negatively correlated with risk of CAD [39] [40]. However, pharmacological studies that increase HDL-C levels have not translated into reduced CAD outcomes [41–43]. Moreover, Mendelian randomization studies have not established a causal relationship between HDL-C metabolism and risk of CAD [44]. However, recent data suggest that when other CAD risk factors are controlled for, HDL-C is associated with an approximate 40% higher risk of CAD [45]. Moreover, it is well-established that not only does HDL plays a key role in RCT but also possesses potent anti-inflammatory and antiatherogenic properties [46]. Therefore, a greater understanding of how the expression of HDL metabolism genes are regulated is important to our overall understanding of risk of vascular disease and CAD.

STAT3 (signal transducer and activator of transcription 3) is a key regulator of numerous important biological processes. Its roles in cancer and immunological disorders are well-established [47, 48]. Importantly, there is emerging evidence that STAT3 plays roles in HDL metabolism, the relationship between inflammation and lipoproteins and, ultimately, in determining CAD risk [49–51]. Hence, our work provides not only an experimentally validated mechanistic link between GWAS signals and enhancers, it also highlights the transcriptional regulation of STAT3 of HDL metabolism genes in determining HDL-C levels.

There are several limitations to our study. First, we have performed enhancer activity validation only in liver-derived cell lines, even though other tissues, such as adipose tissue, also play an important role in cholesterol metabolism. Thus far, other genes uncovered through GWAS of blood lipid traits showed either genotype-specific expression on in liver, but not adipose tissues and/or allele-specific transcription enhancer activity in HepG2 and Huh-7 cells [13, 14]. Additionally, the causal gene for the GWAS signals in our study, namely *ABCA1* and *LCAT*, are expressed in liver and play well-established roles in hepatic cholesterol metabolism. While HepG2 and Huh-7 cells cannot capture all aspects of liver biology, they are commonly used to study liver-specific regulatory mechanisms. Indeed, Huang and Ovcharenko demonstrated that HepG2 enhancers are not only significantly over-represented with liver e-QTLs but also binding sites of liver-specific families of transcription factors, such as HNF4 and FOXA [52]. Importantly, SNPs that are predicted to interrupt HepG2 enhancers are associated with liver–related GWAS findings. Other than these liver-derived cell lines, *in vitro* studies of hepatic gene expression can be performed using hepatocyte-like cells from induced pluripotent stem cells. However, even these cells only capture a fraction of genes expressed in liver and these cells are not amendable to the experimental manipulations required for studies such as ours. Second, we have not tested the enhancers with gene-specific promoters in our *in vitro* assays. Our 3C results showed that the putative enhances are in physical proximity with the promoters, possibly through chromosome looping. However, the allelic difference in transcription activities of these enhancers still need to be determined with the appropriate promoters. Lastly, our ChIP-qPCR and EMSA data showed that *STAT3* interact with specific alleles of the eSNPs. It is not known if higher STAT3 or IL6 expression will enhance the allele-specific enhancer activities. Future studies that use cells that over-express STAT3 endogenously, perhaps through CRISPR-Cas9 transactivation [53], can address this important question.

In conclusion, the GWAS signals for which we have characterized enhancers SNPs have all been identified by independently GWAS efforts and are among the most replicated associations for blood lipids in subsequent meta-analyses. Our study elucidates the underlying mechanism that drives the association signals between 3 clusters of SNPs that are in high LD, with HDL-C. At least one SNP in each cluster is located within an enhancer and demonstrated allele-specific transcriptional activity in a relevant *in vitro* system. We also demonstrated that these 3 enhancers have high likelihood of interacting with the *ABCA1* and *LCAT* promoters by chromatin looping. Binding to the transcription activator, STAT3, plays a role in some cases and the STAT3-enhancer interaction is demonstrated by chromatin immunoprecipitation. Taken together, our results demonstrate that through this approach, the regions that contain GWAS signals, often hundreds of kilobases in size with multiple SNPs serving as statistical proxies to the true functional site, can be narrowed down to much smaller regions and thereby providing experimentally testable hypotheses for the underlying mechanism(s) linking genetic variants to complex traits.

## Supporting information

S1 Fig From GWAS signals to liver-specific eSNPs. Starting from NHGRI catalog of GWAS findings, associated SNPs were first filtered by traits and study population, then expanded to include other SNPs in LD. Sequences containing this set of SNPs were examined for putative enhancer-like epigenetic signature. Putative eSNPs located outside of the predicted promoter region are selected for experimental validation.
(PDF)

S2 Fig Epigenetic landscape around eSNPs. The genomic coordinates around the regions of the eSNPs (hg19) are shown. The chromatin segmentation (ChromHMM) derived from ChIP-Seq data (Broad Institute/ ENCODE Project) in HepG2 cells are shown [11]. Red, orange, yellow, dark green, light green, and blue represent chromatin states predicted to be promoter, strong enhancer, weak enhancers, actively transcribed, weakly transcribed, and insulator, respectively. Putative eSNP locations are indicated by a black line, the putative enhancers tested by a black box, and the 10 kb segments flanking the SNPs are indicated by horizontal bars.
(PDF)

S3 Fig Cell type-specific *in vitro* enhancer activity in HepG2 and HEK 293 cells. Following transient transfection into another hepatoma-derived cell line, HepG2, constructs containing predicted enhancers with rs2675875, rs3847301, and rs1109166 had activity at least comparable to that of a known liver enhancer. In contrast, when the same constructs were transfected into human embryonic kidney cell line, HEK293, two other predicted enhancers containing rs17315646 and rs643531 showed higher transcription activities.
(PDF)

S4 Fig STAT1 and STAT3 binding to rs2575875 alleles *in vitro*. (A) and (B) JASPAR predicted STAT1 and STAT3 preferential binding for rs2575875-A. (C) Competitor oligos for both transcription factors show greater competition against rs2575875-A in EMSA (lanes 5 and 6 versus lanes 11 and 12). TF: transcription factor.
(PDF)

S5 Fig HNF4A binding to rs3847301 alleles *in vitro*. (A) and (B) JASPAR predicted only moderate preference for rs3847301-T, compared to rs3847301-C. (C) HNF4A competitor oligos competed with rs3847301-T and -C for protein binding equally (lane 5 versus lane 10). TF: transcription factor.
(PDF)

S6 Fig Huh-7 and HepG2 genotypes for rs1109166. (A) Huh-7 is homozygous for rs1109166-T (top panel) whereas HepG2 is homozygous for rs1109166-C (bottom panel). (B) Predicted weak STAT3 binding sites of the sequences containing C versus T allele.
(PDF)

S7 Fig IL-6 treatment increased the abundance of nuclear phospho-STAT3 in Huh-7 and HepG2 cell lines. Western blot of Huh-7 and HepG2 nuclear proteins extracted after treatment with rIL-6. The blot was probed with anti-pSTAT3-Y705; followed by stripping and reprobing with anti-total STAT3 anti-HDAC1 antibodies.
(PDF)

S8 Fig Epigenetic landscape of the HDL-C associated region in GALNT2. The putative enhancer region analyzed in this study (“Howard”) contains sequence that demonstrated allele-specific *in vitro* reporter activity in the study by Roman el al. [14].
(PDF)

S1 Table The location of 260 unique GWAS SNPs associated with blood lipid related traits. Location of the SNP is obtained from ANNOVAR, http://wannovar.wglab.org/.
(DOCX)

S2 Table Putative enhancer SNPs and Associations reported by Global Lipids Genetics Consortium (2013). (DOCX)

S3 Table Oligonucleotides used to amplify putative enhancers. (DOCX)

S4 Table Oligonucleotides used in EMSA experiments. (DOCX)

S5 Table Primers used for 3C experiments. (DOCX)

S6 Table Primers used for ChIP experiments. (DOCX)
