# Supplementary figures and images for: Allele-specific enhancers mediate associations between LCAT and ABCA1 polymorphisms and HDL metabolism
Source: PLoS One. 2019 Apr 30;14(4):e0215911. doi: 10.1371/journal.pone.0215911 (PMC6490890; doi:10.1371/journal.pone.0215911)

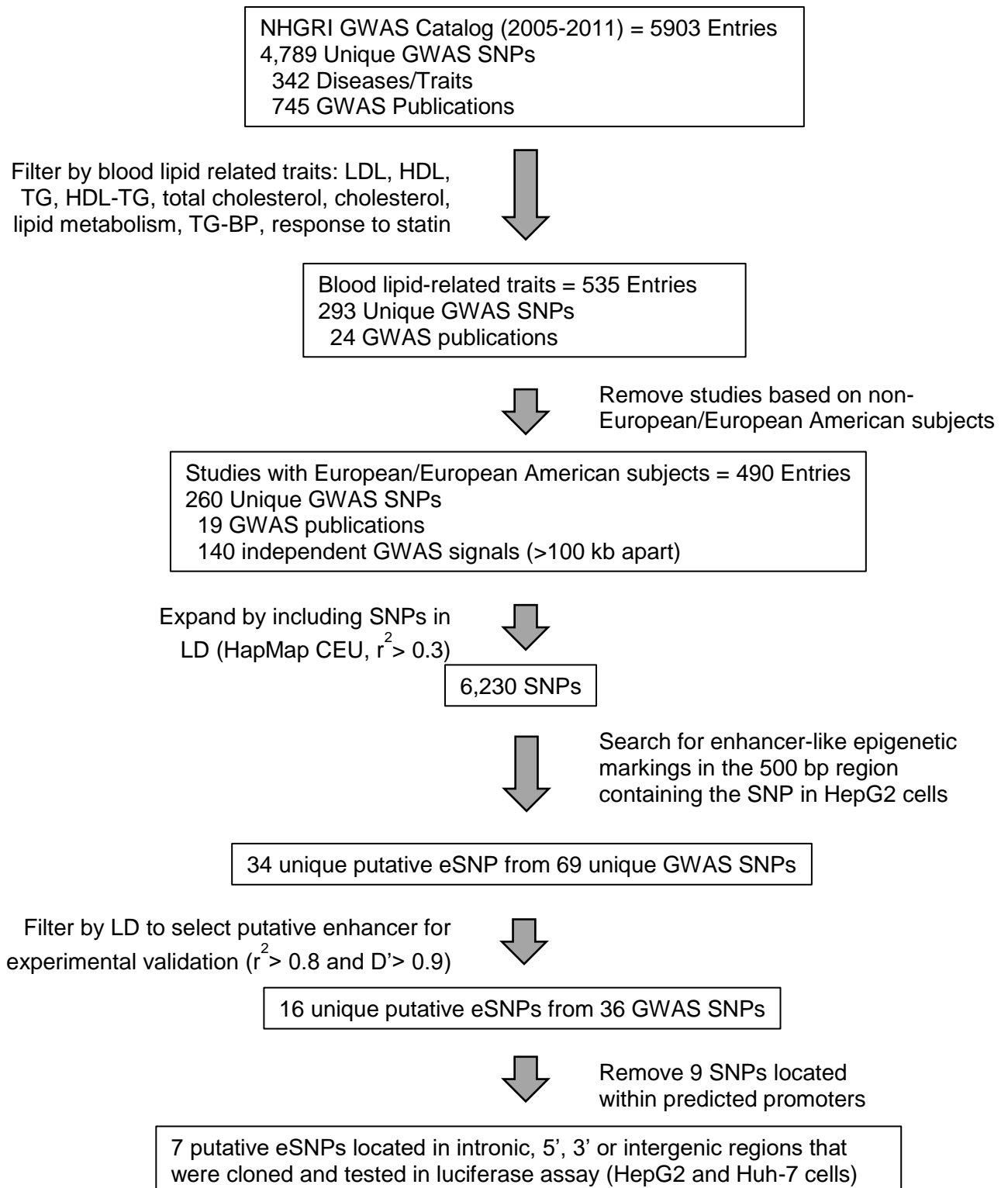

**S1 Fig. From GWAS signals to liver-specific eSNPs.**

Supplement: S1 Fig — Starting from NHGRI catalog of GWAS findings, associated SNPs were first filtered by traits and study population, then expanded to include other SNPs in LD. Sequences containing this set of SNPs were examined for putative enhancer-like epigenetic signature. Putative eSNPs located outside of the predicted promoter region are selected for experimental validation. (PDF) [file pone.0215911.s001.pdf]

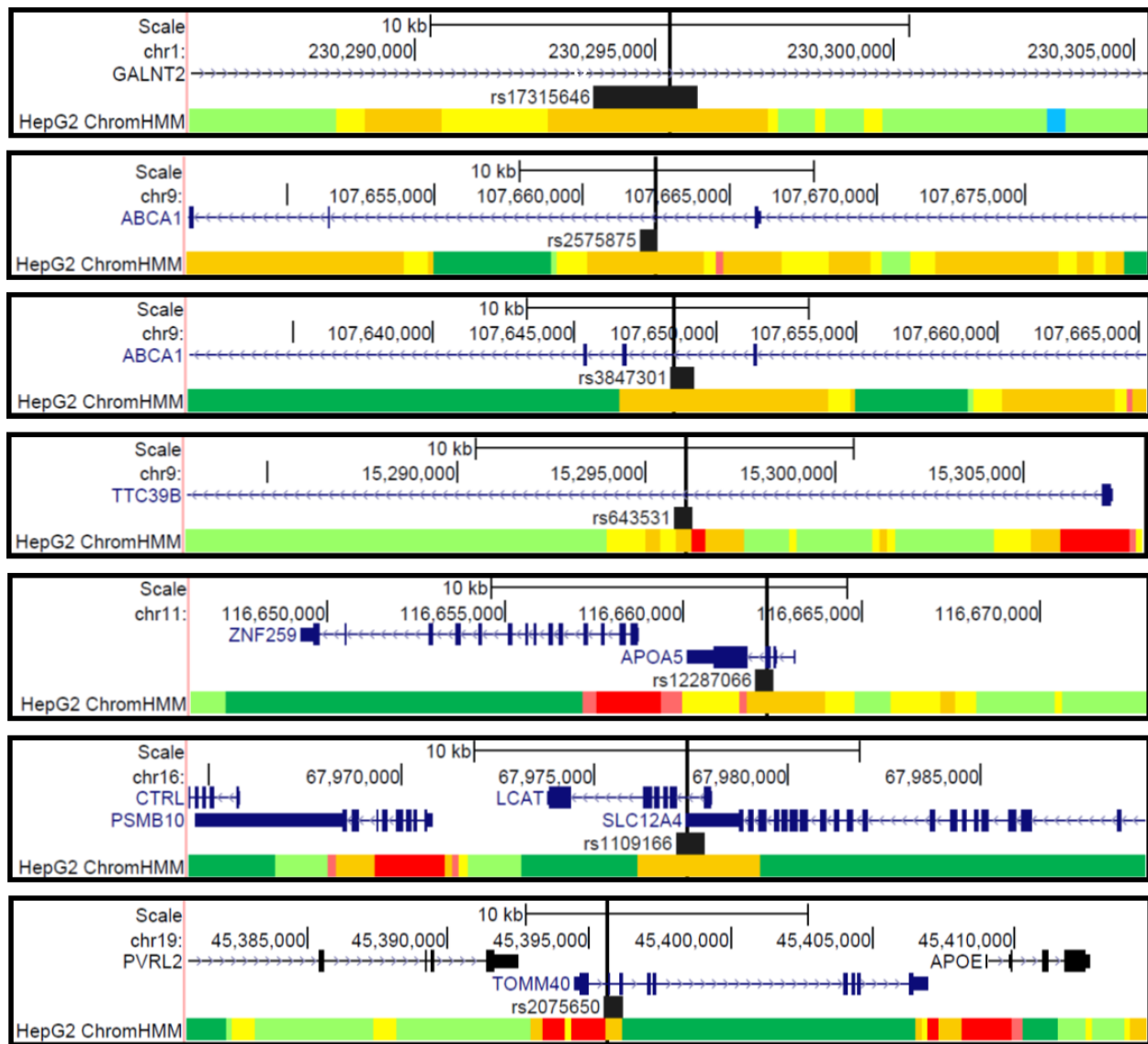

**S2 Fig. Epigenetic landscape around eSNPs.**

Supplement: S2 Fig — The genomic coordinates around the regions of the eSNPs (hg19) are shown. The chromatin segmentation (ChromHMM) derived from ChIP-Seq data (Broad Institute/ ENCODE Project) in HepG2 cells are shown [11]. Red, orange, yellow, dark green, light green, and blue represent chromatin states predicted to be promoter, strong enhancer, weak enhancers, actively transcribed, weakly transcribed, and insulator, respectively. Putative eSNP locations are indicated by a black line, the putative enhancers tested by a black box, and the 10 kb segments flanking the SNPs are indicated by horizontal bars. (PDF) [file pone.0215911.s002.pdf]

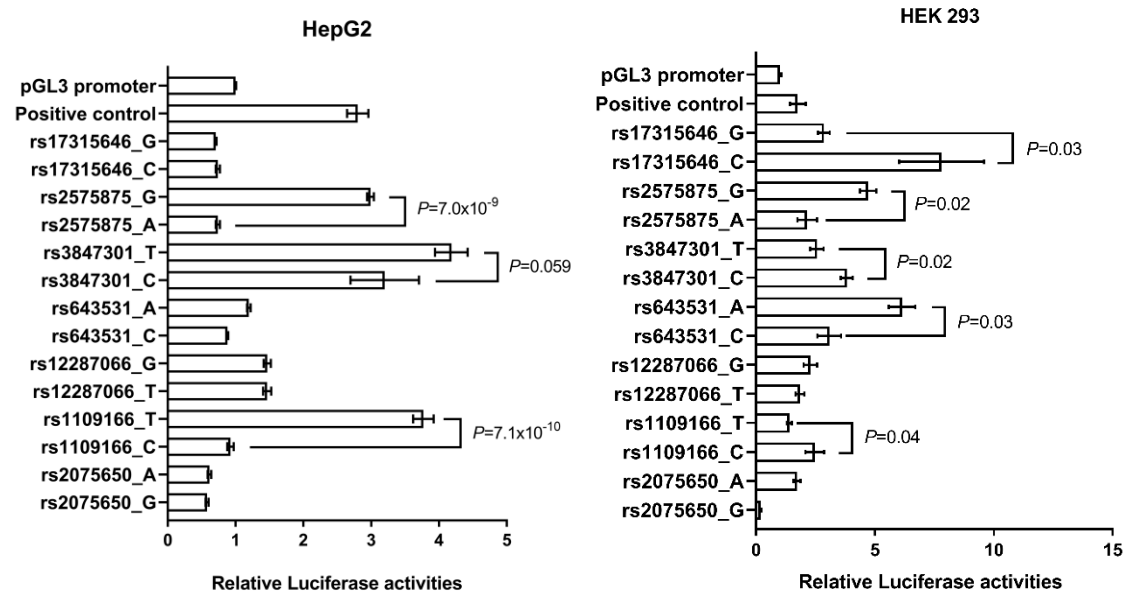

**S3 Fig. Cell type-specific *in vitro* enhancer activity in HepG2 and HEK 293 cells.**

Supplement: S3 Fig — Following transient transfection into another hepatoma-derived cell line, HepG2, constructs containing predicted enhancers with rs2675875, rs3847301, and rs1109166 had activity at least comparable to that of a known liver enhancer. In contrast, when the same constructs were transfected into human embryonic kidney cell line, HEK293, two other predicted enhancers containing rs17315646 and rs643531 showed higher transcription activities. (PDF) [file pone.0215911.s003.pdf]

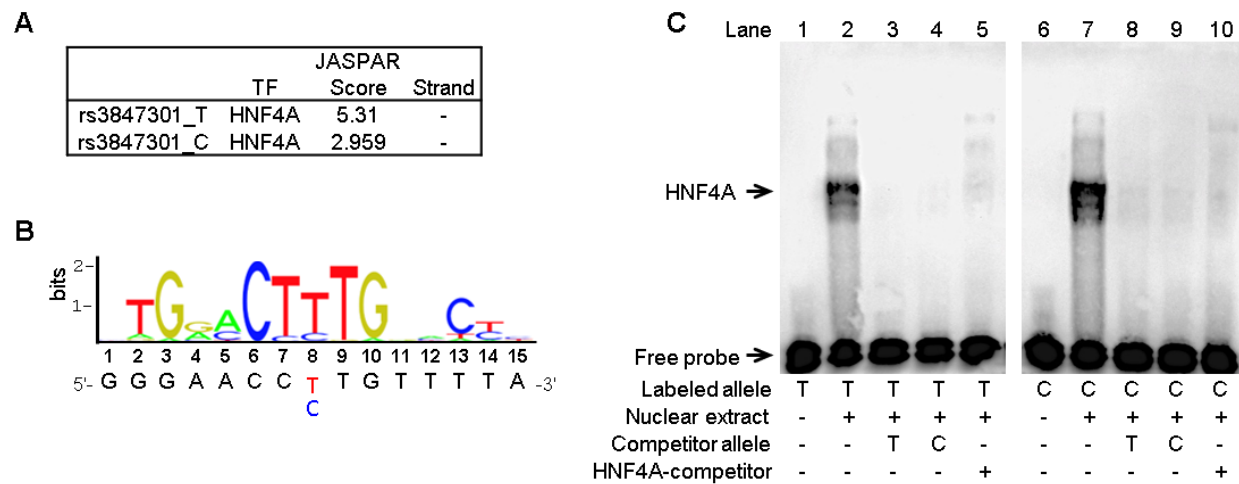

**S5 Fig. HNF4A binding to rs3847301 alleles *in vitro*.**

Supplement: S5 Fig — (A) and (B) JASPAR predicted only moderate preference for rs3847301-T, compared to rs3847301-C. (C) HNF4A competitor oligos competed with rs3847301-T and -C for protein binding equally (lane 5 versus lane 10). TF: transcription factor. (PDF) [file pone.0215911.s005.pdf]

**A**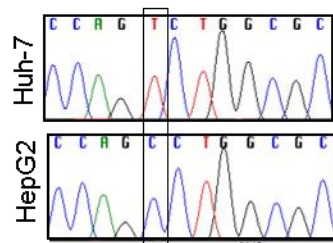**B**

|             | JASPAR |         |        |
|-------------|--------|---------|--------|
|             | TF     | Score   | Strand |
| rs1109166_C | STAT3  | -12.662 | +      |
|             | STAT3  | -16.244 | -      |
| rs1109166_T | STAT3  | -14.168 | -      |

**S6 Fig. Huh-7 and HepG2 genotypes for rs1109166.**

Supplement: S6 Fig — (A) Huh-7 is homozygous for rs1109166-T (top panel) whereas HepG2 is homozygous for rs1109166-C (bottom panel). (B) Predicted weak STAT3 binding sites of the sequences containing C versus T allele. (PDF) [file pone.0215911.s006.pdf]

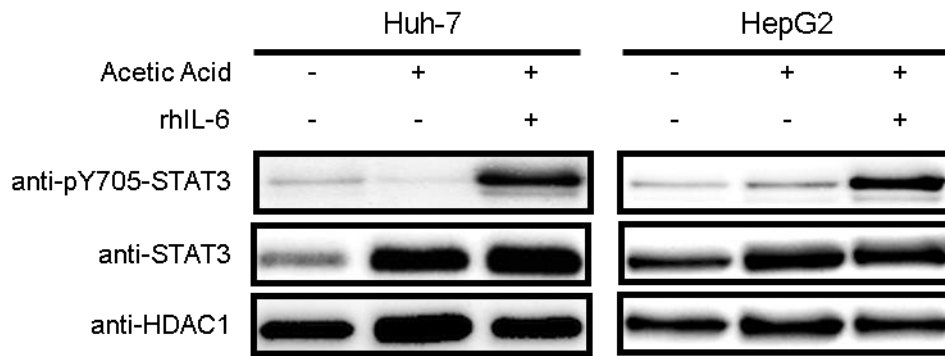

**S7 Fig. IL-6 treatment increased the abundance of nuclear phospho-STAT3 in Huh-7 and HepG2 cell lines.**

Supplement: S7 Fig — Western blot of Huh-7 and HepG2 nuclear proteins extracted after treatment with rIL-6. The blot was probed with anti-pSTAT3-Y705; followed by stripping and reprobing with anti-total STAT3 anti-HDAC1 antibodies. (PDF) [file pone.0215911.s007.pdf]

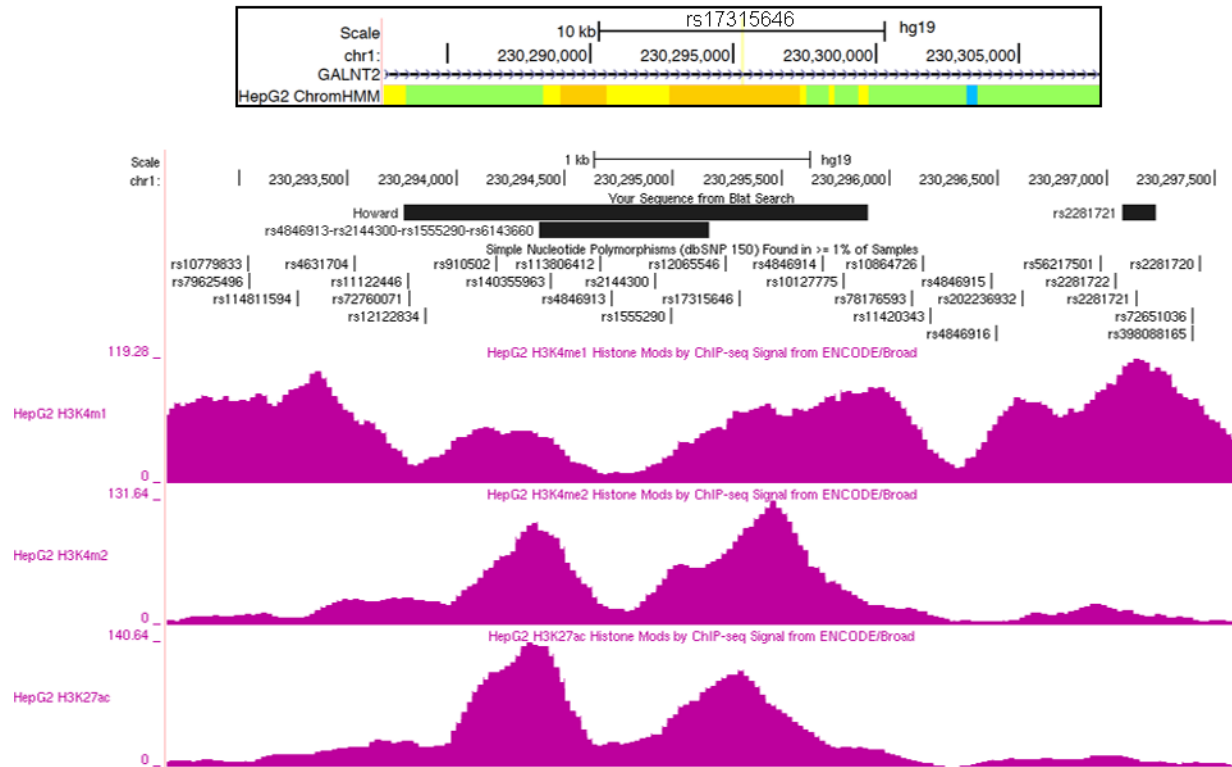

**S8 Fig. Epigenetic landscape of the HDL-C associated region in GALNT2.**

Supplement: S8 Fig — The putative enhancer region analyzed in this study (“Howard”) contains sequence that demonstrated allele-specific in vitro reporter activity in the study by Roman el al. [14]. (PDF) [file pone.0215911.s008.pdf]
