# Supplementary material for: Allele-specific enhancers mediate associations between LCAT and ABCA1 polymorphisms and HDL metabolism
Source: PLoS One. 2019 Apr 30;14(4):e0215911. doi: 10.1371/journal.pone.0215911 (PMC6490890; doi:10.1371/journal.pone.0215911)
Supplement: S4 Fig — (A) and (B) JASPAR predicted STAT1 and STAT3 preferential binding for rs2575875-A. (C) Competitor oligos for both transcription factors show greater competition against rs2575875-A in EMSA (lanes 5 and 6 versus lanes 11 and 12). TF: transcription factor. (PDF) [file pone.0215911.s004.pdf]

A

	TF	JASPAR Score	Strand
rs2575875_G	STAT1	4.377	+
	STAT3	3.326	+
rs2575875_A	STAT1	4.313	-
	STAT1	10.12	+
	STAT3	5.451	-
	STAT3	12.481	+

B

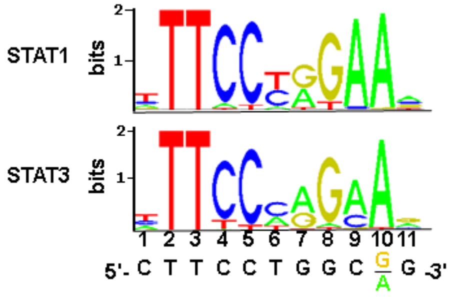

C

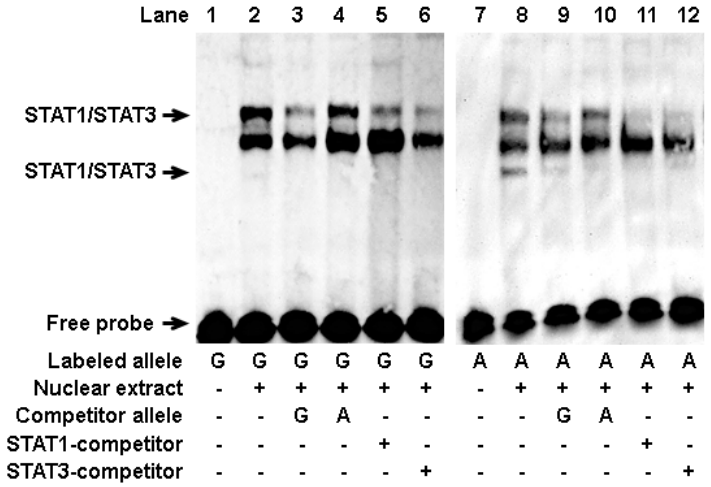

S4 Fig. STAT1 and STAT3 binding to rs2575875 alleles *in vitro*.
