# Supplementary material for: Allele-specific enhancers mediate associations between LCAT and ABCA1 polymorphisms and HDL metabolism
Source: PLoS One. 2019 Apr 30;14(4):e0215911. doi: 10.1371/journal.pone.0215911 (PMC6490890; doi:10.1371/journal.pone.0215911)
Supplement: S1 Table — Location of the SNP is obtained from ANNOVAR, http://wannovar.wglab.org/. (DOCX) [file pone.0215911.s009.docx]

**S1 Table. The location of 260 unique GWAS SNPs associated with blood lipid related traits.**

| **Location** | **Impact on Protein** | **Number of GWAS SNPs** | **Percentage of GWAS SNPs** |
| --- | --- | --- | --- |
| downstream |  | 9 | 3.5% |
| exonic |  | 19 | 7.3% |
|  | missense | (13) | (5.0%) |
|  | synonymous | (5) | (1.9%) |
|  | stopgain | (1) | (0.4%) |
| intergenic |  | 92 | 35.4% |
| intronic |  | 113 | 43.5% |
| ncRNA_intronic |  | 7 | 2.7% |
| ncRNA_exonic |  | 1 | 0.4% |
| upstream |  | 4 | 1.5% |
| UTR3 |  | 15 | 5.8% |
| **Total** |  | 260 | 100.0% |

Location of the SNP is obtained from ANNOVAR, http://wannovar.wglab.org/
