# Supplementary material for: Allele-specific enhancers mediate associations between LCAT and ABCA1 polymorphisms and HDL metabolism
Source: PLoS One. 2019 Apr 30;14(4):e0215911. doi: 10.1371/journal.pone.0215911 (PMC6490890; doi:10.1371/journal.pone.0215911)
Supplement: S2 Table — (DOCX) [file pone.0215911.s010.docx]

**S2 Table. Putative enhancer SNPs and association signals reported by Global Lipids Genetics Consortium.**

| Chr | eSNP | Nearest gene | Trait(s) | Global Lipids Genetics Consortium | | | | |
| --- | --- | --- | --- | --- | --- | --- | --- | --- |
|  |  |  |  | GWAS SNP | Alleles minor/major | MAF | Effect size | Joint P-value |
| 1 | rs17315646 | *GALNT2* | HDL  TG | rs4846914 | G/A | 0.41 | -0.048  0.04 | 4x10^-41^  7x10^-31^ |
| 9 | rs2575875 | *ABCA1* | HDL  TC | rs1883025 | T/C | 0.25 | -0.07  -0.067 | 2x10^-65^  6x10^-53^ |
| 9* | rs3847301 | *ABCA1* | HDL | rs3890182 | T/C | 0.13 | -0.01 | 3 × 10^−10^ |
| 9 | rs643531 | *TTC39B* | HDL  TC | rs581080 | G/C | 0.21 | -0.042  -0.038 | 1x10^-19^  1x10^-13^ |
| 11 | rs12287066 | *APOA1* | TG  TC  HDL  LDL | rs964184 | C/G | 0.16 | -0.234  -0.121  0.106  -0.086 | 7x10^-224^  3x10^-55^  6x10^-48^  2x10^-26^ |
| 16 | rs1109166 | *LCAT* | HDL | rs16942887 | A/G | 0.14 | 0.083 | 8x10^-54^ |
| 19 | rs2075650 | *APOE* | LDL  TC  HDL | rs4420638 | G/A | 0.19 | 0.225  0.197  -0.067 | 2x10^-178^  1x10^-149^  2x10^-21^ |

TG, triglycerides; TC, total cholesterol; MAF, minor allele frequency. Effect sizes are given with respect to the minor allele in s.d. The trait corresponding to the strongest P value is listed first. * Since the Global Lipids Genetic Consortium reported only 1 GWAS signal per gene (Nat Genet. 2013 Nov;45(11):1274-1283), effect size and p-value of eSNP/GWAS SNP rs3847301/rs3890182 are extracted from another study (Nat Genet. 2008 Feb;40(2):189-97).
