# Supplementary material for: Allele-specific enhancers mediate associations between LCAT and ABCA1 polymorphisms and HDL metabolism
Source: PLoS One. 2019 Apr 30;14(4):e0215911. doi: 10.1371/journal.pone.0215911 (PMC6490890; doi:10.1371/journal.pone.0215911)
Supplement: S3 Table — (DOCX) [file pone.0215911.s011.docx]

**S3 Table. Oligonucleotides used to amplify putative enhancers.**

| **Primer** | **Sequences (5' to 3')** | **Genomic location of amplicon (build 37­)** |
| --- | --- | --- |
| rs643531_For | AGGAAATGGGAAGCCAGAAT | chr9:15,295,768-15,296,221 |
| rs643531_Rev | TCTGAGATAGAAAACCAACTTGAGC |  |
| rs1109166_For | GAGCAGCAGCCCTCACTCT | chr16:67,977,120-67,977,862 |
| rs1109166_Rev | CCGTCATCCTCGGTAAGC |  |
| rs2075650_For | CAGGTGGAATCCTTTGTGAGA | chr19:45,395,525-45,396,198 |
| rs2075650_Rev | AAGGAAGCTCACCTCTGTGG |  |
| rs2575875_For | TTTTCCCAGCCACATGACTA | chr9:107,662,000-107,662,572 |
| rs2575875_Rev | GCCCATTAGTTTGCAGGTCT |  |
| rs3847301_For | GGCACTGTGGGAGTGAATCT | chr9:107,648,418-107,649,235 |
| rs3847301_Rev | ACCCTCCAGGGACCTAGTTG |  |
| rs10202854_For | CGAAATCCATGTCAGGGAAC | chr2:169,047,193-169,047,583 |
| rs10202854_Rev | ACAGGTGGCTTGAGGTTGAA |  |
| rs12287066_For | TCCTTTGATTCTGGGGACTG | chr11:116,662,022-116,662,511 |
| rs12287066_Rev | GACTTCAACGTGGGGGTGT |  |
| rs17315646_For | AAGCCAAGTCAGGGAAGTGA | chr1:230,293,757-230,295,900 |
| rs17315646_Rev | ACAATGGGTTGGAGAAGCAC |  |
| Positive Control Enhancer _For | CCTCTGGGCACACGGACTCT | chr15:96,808,114-96,808,684 |
| Positive Control Enhancer _Rev | CTAATAAAGTCCAAGGGTC |  |
