# Supplementary material for: Allele-specific enhancers mediate associations between LCAT and ABCA1 polymorphisms and HDL metabolism
Source: PLoS One. 2019 Apr 30;14(4):e0215911. doi: 10.1371/journal.pone.0215911 (PMC6490890; doi:10.1371/journal.pone.0215911)
Supplement: S4 Table — (DOCX) [file pone.0215911.s012.docx]

**S4 Table. Oligonucleotides used in EMSA experiments.**

| **Primer** | **Forward (5' to 3')** | **Reverse (5' to 3')** |
| --- | --- | --- |
| rs1109166T/A | AGAGCAAACACCCAG**T**CTGGCGCTCCTTTA | TAAAGGAGCGCCAG**A**CTGGGTGTTTGCTCT |
| rs1109166C/G | AGAGCAAACACCCAG**C**CTGGCGCTCCTTTA | TAAAGGAGCGCCAG**G**CTGGGTGTTTGCTCT |
| rs2575875G/C | TCTAGTCTTCCTGGC**G**GCAGCACACAGGCT | AGCCTGTGTGCTGC**C**GCCAGGAAGACTAGA |
| rs2575875A/T | TCTAGTCTTCCTGGC**A**GCAGCACACAGGCT | AGCCTGTGTGCTGC**T**GCCAGGAAGACTAGA |
| rs3847301T/A | TCCAAAGTGGGAACC**T**TGTTTTATTCATCT | AGATGAATAAAACA**A**GGTTCCCACTTTGGA |
| rs3847301C/G | TCCAAAGTGGGAACC**C**TGTTTTATTCATCT | AGATGAATAAAACA**G**GGTTCCCACTTTGGA |
| STAT3 competitor | GATCCTTCTGGGAATTCCTAGATC | GATCTAGGAATTCCCAGAAGGATC |
| STAT1 competitor | CATGTTATGCATATTCCTGTAAGTG | CACTTACAGGAATATGCATAACATG |
| HNF4A competitor | CTCAGCTTGTACTTTGGTACAACTA | TAGTTGTACCAAAGTACAAGCTGAG |
