# Supplementary material for: Allele-specific enhancers mediate associations between LCAT and ABCA1 polymorphisms and HDL metabolism
Source: PLoS One. 2019 Apr 30;14(4):e0215911. doi: 10.1371/journal.pone.0215911 (PMC6490890; doi:10.1371/journal.pone.0215911)
Supplement: S5 Table — (DOCX) [file pone.0215911.s013.docx]

**S5 Table. Primers used for 3C experiments.**

| **Potential 3C Interaction** | **Primer** | **Sequences (5' to 3')** | **Genomic location of primer (build 37­)** |
| --- | --- | --- | --- |
| LCAT rs1109166 SNP-IL6 responsive element  (PstI) | LCAT_IL-6_element | CTATCTCCTGCAATTGTCCTCAG | chr16:67,979,457-67,979,479 |
|  | rs1109166_enhancer_Pst | CTATCTGTTCCCACCTTGGACTT | chr16:67,977,403-67,977,425 |
| LCAT promoter-rs1109166 containing enhancer  (MspI) | rs1109166_enhancer_outer | GTCTGGTCACTGCAGCATCTGGGGTGAC | chr16:67,977,172-67,977,199 |
|  | rs1109166_enhancer_inner | GAATCCAGAGTCCAGAGTGAGGGCTGCTGCTC | chr16:67,977,120-67,977,151 |
|  | LCAT_promoter | ACTCCCACACCAGATAAGGACAGCCCAGTG | chr16:67,978,038-67,978,067 |
| ABCA1 promoter- rs2575875 containing enhancer  (NcoI) | ABCA1_promoter | CTTCAAGAAGAGAATGGGTAGTATATACAC | chr9:107,688,469-107,688,498 |
|  | rs2575875_enhancer | GTTAGGCAAAGAATAAATTGAATTGAGTTG | chr9:107662267-107,662,296 |
| ABCA1 promoter- rs3847301 containing enhancer  (PstI) | ABCA1_promoter | GACGATGTCCTAGTACGAGCTCTGGTGA | chr9:107,690,033-107,690,060 |
|  | rs3847301_enhancer_outer | GATTAACTTCTCAGCCCATTTGCATGAA | chr9:107,648,541-107,648,568 |
|  | rs3847301_enhancer_inner | CGATGCTCACCACAGCTCTGTGAAGTTAAT | chr9:107,648,304-107,648,333 |
