# Supplementary material for: Allele-specific enhancers mediate associations between LCAT and ABCA1 polymorphisms and HDL metabolism
Source: PLoS One. 2019 Apr 30;14(4):e0215911. doi: 10.1371/journal.pone.0215911 (PMC6490890; doi:10.1371/journal.pone.0215911)
Supplement: S6 Table — (DOCX) [file pone.0215911.s014.docx]

**S6 Table. Primers used for ChIP experiments.**

| **Primer** | **Sequences (5' to 3')** | **Genomic location of primer (build 37­)** |
| --- | --- | --- |
| rs1109166 qPCR_For | CTGCTTACACCCCCTCTCC | chr16:67,977,256-67,977,275 |
| rs1109166 qPCR_Rev | TCTGTTCCCACCTTGGACTT | chr16:67,977,403-67,977,422 |
| rs2575875qPCR_For | CCAGGCTGAACATAGCGTTT | chr9:107,662,460-107,662,479 |
| rs2575875qPCR_Rev | AGGCCCATTAGTTTGCAGGT | chr9:107,662,555-107,662,574 |
| GAPDH_For | CTTCGTATGACTGGGGGTGT | chr12:6,645,430-6,645,449 |
| GAPDH_Rev | GGCCCAAGAGGTTGAATTTT | chr12:6,645,524-6,645,543 |
